# Patients’ views about parathyroid transplantation for post-thyroidectomy hypoparathyroidism

**DOI:** 10.1007/s00423-018-1693-y

**Published:** 2018-07-03

**Authors:** Alexander Stevenson, Radu Mihai

**Affiliations:** 1 Oxford University Hospitals NHS Foundation Trust, Oxford, UK; 2 Blenheim Head & Neck Unit, Churchill Cancer Centre, Old Road, Headington, Oxford, OX3 7LE UK

**Keywords:** Hypoparathyroidism, Post-thyroidectomy, Parathyroid transplantation

## Abstract

**Background:**

Permanent hypoparathyroidism (hypoPT) represents the most common postoperative complication associated with total thyroidectomy. Current treatment relies on high-dose calcium and/or vitamin D supplementation, but often this is insufficient and some patients remain symptomatic. Parathyroid allotransplantation is a new therapeutic option described recently in the literature. This study aims to investigate the patients’ acceptability of parathyroid transplantation as a potential new treatment for hypoPT.

**Method:**

Online survey of members of *HypoParaUK*, a support group for individuals affected by hypoPT.

**Results:**

Responses were received from 252 hypoPT patients. Majority declared to experience severe symptoms despite regular medical treatment. On a severity scale of 0–5, symptoms that were most troublesome were fatigue (3.8), low sense of well-being (3.5), and numbness/tingling (2.9). On a scale of 0–10, on average, their current quality of life (QoL) was 5 ± 3 and they expected this would improve to 7 ± 2 with correction of their hypoPT. Forty-four percent of patients were extremely interested in a potential technique involving intramuscular injection of parathyroid cell suspension compared to just 14% who were interested in the more invasive procedure of implantation of a parathyroid allograft into the forearm. The main concerns expressed were related to the possible need for immunosuppressive therapy.

**Conclusion:**

Patients with severe symptomatic hypoPT seem interested to consider participation in a clinical trial exploring the feasibility and success rate of parathyroid transplantation.

**Electronic supplementary material:**

The online version of this article (10.1007/s00423-018-1693-y) contains supplementary material, which is available to authorized users.

## Introduction

Primary hypoparathyroidism (hypoPT) is defined as a low calcium levels in a patient with low/absent parathyroid hormone (PTH) levels. Its prevalence was estimated as 37 per 100,000 person-years in the USA and 22 per 100,000 person-years in Denmark [1]. Such data are currently lacking in most other countries.

The most common cause of hypoPT is injury to the parathyroid glands during bilateral thyroid surgery. The likely mechanism of injury is through compromising their vascular supply, thermic injury during dissection or inadvertent resection [2]. The importance of preserving well-vascularized glands has been reconfirmed by recent studies using indocyanine green fluorescence angiography that showed PTH levels on postoperative day 1 were normal in all patients who had at least one well vascularized parathyroid gland and none of these patients required treatment for hypoPT [3]. In addition, inadvertent removal of the parathyroid glands during thyroid surgery is reported to occur in as little as 6% of patients [4] or as high as 25% [2, 5]. More recently, intraoperative infrared imaging has been used to identify parathyroid glands based on their autofluorescence [6] but it is yet to be demonstrated whether routine use of this technique will decrease the risk of postthyroidectomy hypoPT.

When inadvertent removal of parathyroid glands is identified intraoperatively, such glands are reimplanted in a muscle pocket with the expectation that this would favor the recovery of parathyroid function. In contrast, several authors found that autotransplantation does not influence the rate of postoperative hypoPT [7] and that the prevalence of parathyroid failure after total thyroidectomy was similar whether a parathyroid gland was inadvertently excised or autotransplanted [8]. The current view is that in situ parathyroid preservation of parathyroid glands is critical in preventing permanent hypoPT after total thyroidectomy [9].

Injuries to the parathyroid glands during thyroid surgery lead to a drop in PTH levels within hours and to hypocalcaemia within 24–48 h after the operation. The initial signs of postoperative hypocalcaemia are expected to occur in up to 30% of patients undergoing total thyroidectomy. Once hypocalcaemia is identified on routine postoperative bloods or when patients develop symptoms they are offered calcium supplements with or without vitamin D supplements. Within 4–6 weeks after the operation, about two thirds of cases need no further treatment (*transient hypoPT*) and a third of patients will still require treatment (*protracted hypoPT*). In long term, about a quarter of patients with protracted hypoPT will develop permanent hypoPT. A prospective, cross-sectional study of 3605 patients operated in five academic hospitals by 22 participating surgeons found no correlation between immediate and permanent hypoPT [10] as most patients have only transient hypoPT. Slow recovery of hypoPT has been reported to occur in up to 2 years after surgery [11] hence regular biochemical assessment is necessary in such patients.

Little is known about the impact of hypoPT on quality of life (QoL) and there is no specific QoL questionnaire developed for this condition. A recent literature review highlighted that most studies did not report the incidence of disability, sick leave, QoL, nor the occurrence of cramps, tetany or seizures [12]. Furthermore, the combination of postsurgical hypothyroidism and hypoPT might both contribute to impaired QoL [13]. Unexpectedly, a recent analysis of nearly 500 patients recorded on the Scandinavian Quality Register for Thyroid, Parathyroid and Adrenal Surgery showed that the risk of death was significantly higher among patients with permanent hypoPT after total thyroidectomy (adjusted hazard ratio 2·09, 95%CI1·04 to 4·20) [14].

Medical management of hypoPT has been reviewed in recent guidelines issued by the European Society of Endocrinology [15]. The use of oral calcium and vitamin D supplements cannot resolve all problematic aspects of the disease, such as abnormal bone remodeling and reduced QoL, and such treatment is associated with long-term complications, including nephrolithiasis, nephrocalcinosis, renal impairment, cataracts and cerebral calcifications. Though the use of recombinant PTH can normalize biochemical parameters [16], there is no data on its impact on QoL.

In the context of imperfect management options, patients’ groups offer invaluable support to people affected by this condition and one such group functions in the UK (*HypoParaUK*). Because medication fails to prevent symptoms of hypocalcaemia in some patients, new approaches are being sought by patients and clinicians alike. A recent case report of a 32-year-old female with intractable hypoPT who underwent successful allotransplantation of two parathyroid glands in the forearm [17] triggered renewed hopes of many members of *HypoParaUK* group who find current treatment options unsatisfactory. As a consequence, this study aimed to explore the views of patients with hypoPT related to the severity of their symptoms and the development of parathyroid transplantation as a potential new therapy.

## Methods

An online survey was created (https://www.surveymonkey.co.uk/r/ParaTransplant). The invitation letter was circulated by the President of the HypoParaUK group to all registered members in February 2015 and the survey stayed opened until September 2015. All replies were received within 6 weeks after sending the invitation to participate. The survey remained opened for 12 weeks before replies were analyzed.

The full list of questions appears in Appendix 1. The first section of the survey explored the severity of frequency of symptoms experienced by individual patients and their impact on quality of life. The second section of the survey explored their views about potential techniques that could be developed. Even though none of these techniques are in current clinical practice, three possible options were described together with their main potential advantages and disadvantages (Table 1).

**Table 1:** Potential scenarios for parathyroid transplantation

	Description	Advantages	Disadvantages
Option 1	Intramuscular injection of cell suspension from a parathyroid gland removed from another patient	Simple technique, potentially not requiring long-term immunosuppressants	Potentially unreliable
Option 2	Small incision over the forearm muscles to create a pocket in which to place fragments of a parathyroid gland removed from another patient	Acceptably simple operation	Very likely to need long-term immunosuppressant therapy
Option 3	Creation of a graft formed by growing cells from a parathyroid gland into a patch of skin from the same patient, followed by a transplantation of this patch of skin over your forearm	Ability to check that the graft produces parathyroid hormone before being transplanted	Certain need for long-term immunosuppressant therapy

Average values and ranked values were automatically calculated by the website’s software. Individual values could not be downloaded hence no comparison could be made between paired-samples . For the same reason, it was not possible to compare the replies of patients with postthyroidectomy hypoPT from those of patients with other causes of hypoPT.

## Results

The survey was emailed to 1128 patients members of *HypoParaUK* group, of whom 494 opened the email. Replies were received from 252 patients. They had a diagnosis of hypoPT for a median of 5 years (range 1 year to over 10 years), with the majority having had thyroid surgery (*n* = 169, 69%) and a minority other causes of hypoPT (post parathyroidectomy, *n* = 24; idiopathic *n* = 39; genetic, *n* = 12). As the identity of HypoParaUK members remains confidential, there was no effort made to enquire about gender and age. The age of the patients is not being collected by the HypoParaUK group but it is known to the group organizers that the majority of their members are between 20 and 60.

**Fig. 1:**
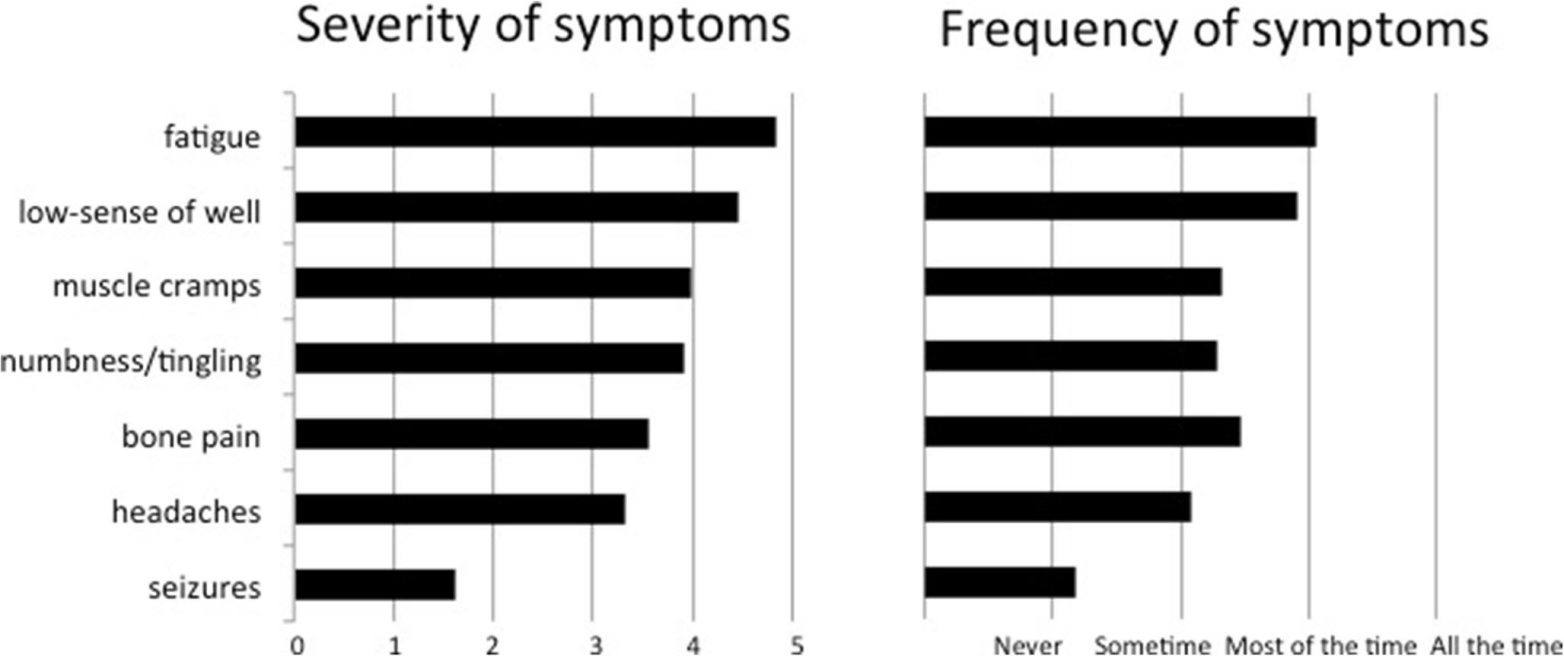
The severity and frequency of symptoms of patients with permanent hypoparathyroidism. Severity of symptoms was rated on a scale of 0–5 (least severe–most severe). Frequency of symptoms was rated on a scale of 1–4 with 1 = never, 2 = sometime, 3 = most of the time, 4 = all the time

The severity and frequency of symptoms are summarized in Fig. 1. On a scale 1–5, highest average scores for severity of symptoms were 4.8 ± 1.8 for fatigue and 4.5 ± 1.9 for low sense of well-being. These were also the most commonly experienced symptoms. Only 4% of patients never experienced fatigue and numbness/tingling sensation while 30–35% experienced these symptoms almost all the time (Fig. 1). In addition, 149 patients listed a myriad of associated symptoms rarely described in literature.

The majority of patients were taking regular calcium supplements (*n* = 179) and/or vitamin D supplements (*n* = 210). Despite regular medications, 93% of responders needed emergency admission to hospital within the last 6 months and two thirds of patients declared that the symptoms interfered with their job and family life. Sixty-one percent of patients described their current health to be fair or poor and 35% of them felt that their current health is worse than 1-year previously. Vigorous activities such as running, heavy lifting objects, or participating in strenuous sports were deemed very limited by a third of the patients.

**Fig. 2:**
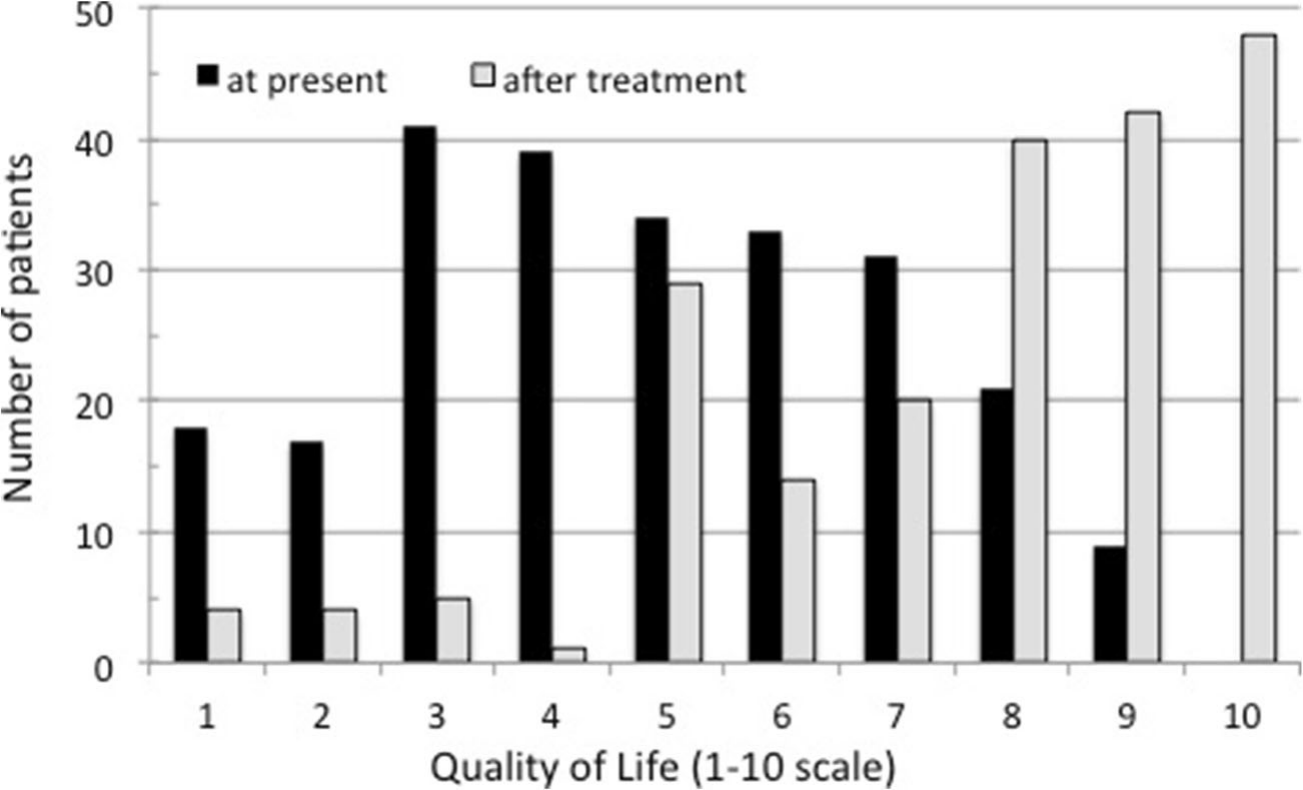
Estimated quality of life in patients with hypoparathyroidism. Quality of life was quantified on a 1–10 scale (1 = worst QoL, 10 = best QoL). Participants were asked about their current QoL (full bars) and their expected QoL after a potentially functional paragrathyroid graft (empty bars)

Quality of life (QoL) estimated on a 10-point visual analogue scale (10 corresponding to perfect life without any medical complaints and 1 corresponding to no QoL). Ranked average was significantly different between current QoL (4.7 ± 2.1) and expected QoL after correction of hypoPT 7.7 ± 2.2 (Fig. 2). There was no possibility to match the responses of individual patients hence no statistical analysis could be made betweed paired samples. For the entire cohort, the median values for current QoL and expected QoL were vastly different (5 vs. 8) and the lower quartile of the population had a current score of 3 and expected to have a score of 7 after a successful parathyroid graft.

Participants were asked about a parathyroid transplant procedure which could restore their parathyroid function and normalize calcium levels, freeing them of symptoms; however, the procedure would necessitate taking immunosuppressive medications which could reduce their lifespan. On average, patients declared to be ready to give up an average of 3.6 years (median 1 year) of their remaining life to undergo the procedure providing they should have a median life expectancy of at least 11 years. If anti-rejection medications reduced their lifespan by one third, the minimum acceptable level of improvement in quality of life was on average 80%. The mean maximum chance of rejection within 1 year that participants would accept was 36%. After explanation of the potential side-effects of immunosupp, 31% of patients would still want to get a parathyroid transplant.

**Fig. 3:**
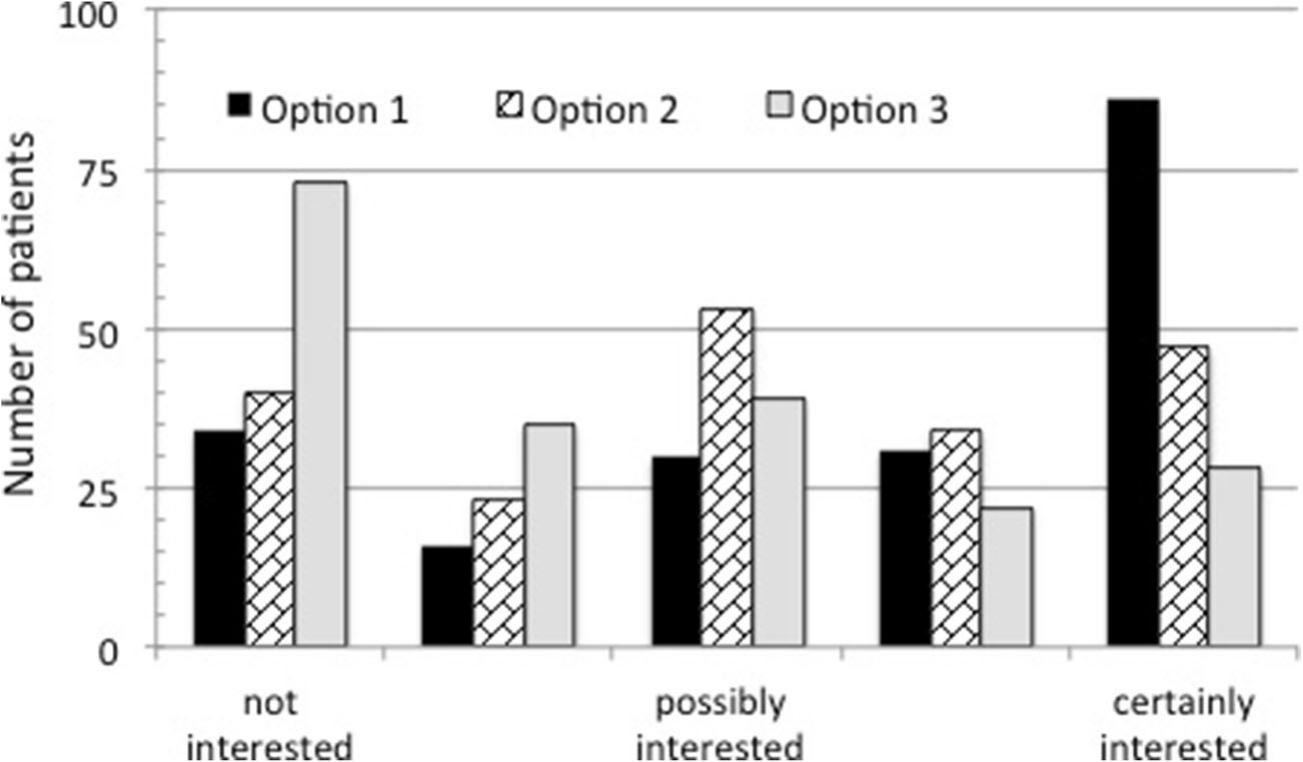
Acceptability of different techniques for parathyroid transplantation. The responses of participants asked to score their interest in three potential techniques of parathyroid transplantation

Three possible scenarios for parathyroid transplantation were described in order to explore patients’ views about their feasibility and risk/benefit balance (Table 1). They were aware that none of these treatments are available and their development would require extensive and timely clinical work. Majority of patients were very interested in Option 1 (Fig. 3). The main concerns regarding Options 2 and 3 were related to the use of immunosuppressant medication. Overall two thirds of patients volunteered to be involved in the future development of such a project either by being considered as potential candidates or by providing support to patients in a similar situation.

## Discussion

Postoperative hypoPT represents the most common complication of thyroid surgery. Its impact on patients’ quality of life (QOL) is significantly underestimated by surgeons. In a large study including 340 postsurgical patients with permanent hypoPT, 200 controls and 102 surgeons, 47% of patients believed that their health was “much worse” than before surgery, compared with 16% of surgeons and 7% of controls. HypoPT patients also reported far more negative effects on QOL, from interference with social activities, paresthesias, muscle cramping, and medications than were not anticipated by surgeons or controls [18]. Similarly, in a study of 522 patients from Norway, patients with postsurgical hypoPT scored worse than those with nonsurgical-hypoPT when compared with normative population on SF-36 Health Survey and Hospital Anxiety and Depression scale [19].

The recent guidelines for the European Society of Endocrinology based on a systematic review of 1100 articles concluded that little evidence is available on how best to treat hypoPT [15]. Data on QoL and the risk of complications have just started to emerge and clinical trials on how to optimize therapy are essentially non-existent. Most studies are of limited sample size, hampering firm conclusions [15]. Recombinant PTH was reported to improve both the physical and mental component scores of the SF-36 Health Survey within 2 months and remain stable during 5 years treatment in a group of 69 patients [20] but this medication is seldom available and the response is variable between patients.

Exploring patients’ views about the need for exploring new therapies is deemed a crucial component of the process for obtaining ethical approval and funding opportunities. Engagement of patients groups is encouraged by all funding bodies. The options explored were deemed feasible (as suggested by a few case reports) and clinically reasonable. The high interest expressed by the majority of respondents could be biased by the fact that most participants had very severe symptomatic hypoPT, with 93% of them having had an emergency admission for severe hypocalcaemia during the preceding 6 months, hence their views about the need for a more radical treatment would be different than those of patients with minimally symptomatic disease.

The most recent view is that only the number of parathyroid glands left in situ correlate with normocalcaemia. A study of 657 patients undergoing total thyroidectomy showed that prevalence of hypocalcaemia and of protracted/permanent hypoPT was inversely correlated to the number of glands left in-situ (16% of cases with one or two glands compared to 2.5% when four glands were left in-situ) [9]. Furthermore, there is increased awareness that autotransplantation of inadvertently removed parathyroid glands during thyroid surgery might not safeguard against the risk of hypoPT. Traditionally the belief was that small fragments of parathyroid gland(s) placed in a muscular pocket will regain their vascular supply and will return to a degree of functional ability but clinical data to reinforce this practice was scarce. One group reported that parathyroid gland re-implantation in forearm subcutaneous/muscle tissue during thyroid surgery leads to recovery of graft function in 48% of patients at 1 week, and 96% of patients at 3 months after surgery [21]. In a separate study, the efficacy of two techniques for autotransplantation after total parathyroidectomy for secondary renal hyperparathyroidism was compared. For the implant group (*n* = 31), a parathyroid gland was divided into 10–12 pieces (each of 2-mm in diameter) before embedding into the deltoid or brachioradialis muscle. Patients in the inject group (*n* = 35), each had a finely minced gland injected into the deltoid. Graft function was achieved in 87% of implant patients and 69% of inject patients [22].

Irrespective of the operative expertise, intraoperative maneuvers and postoperative medical management, some patients develop permanent hypoPT and become very distressed by the symptoms associated with this condition. Our study demonstrates both a significant impairment in the QoL and lack of effectiveness of current treatment strategies in a number of those affected by hypoPT. For a minority of patients, it could be that allotransplantation might be the only effective treatment but the feasibility of such an approach has been addressed only in a very small number of isolated case reports.

Retrieval of parathyroid glands from healthy donors was described in a case report of a 32-year-old female with intractable persistent hypocalcemia after neck surgery for papillary thyroid cancer who underwent parathyroid living-donor allotransplantation of two healthy parathyroid glands to the recipient’s left forearm. Nearly 3 years after transplantation, the patient remained asymptomatic with normal serum levels of calcium and PTH [17].

In a cohort study, parathyroid cell suspensions obtained from four donors who had undergone subtotal parathyroidectomy for secondary hyperparathyroidism in chronic renal failure were transplanted in 10 patients with permanent hypoPT after short-term cell cultivation. Prednisolone were used as immunosuppressant for the first 10 days and discontinued thereafter. Allograft function was observed in 7 patients (70%) at a mean follow-up of 12 months. Daily oral calcium and vitamin D supplementations discontinued totally in 7 patients and no complications were observed [23].

A second cohort study described the treatment of 85 patients with iatrogenic hypoPT using cultured parathyroid cell suspensions from donors undergoing parathyroidectomy for secondary/tertiary parathyroidism. The cell suspension was injected into the skin and subcutaneous tissue of the non-dominant forearm with no immunosuppressive therapy commenced. The mean cellular allograft survival was 6 months, with 55% of patients retaining at least 2 months of allograft endocrine function [24].

A number of case reports described parathyroid allotransplantation in renal transplant patients already receiving immunosuppressive therapy. These patients develop tertiary hyperparathyroidism for which parathyroidectomy is a recognized treatment and as a result they can develop hypoparathyroidism. One patient with severe, symptomatic refractory hypoPT was treated successfully with two parathyroid allografts and remained clinically asymptomatic 8 months later [25]. A second renal transplant patient who was hypoPT secondary to a previous subtotal parathyroidectomy had a parathyroid allograft inserted into his left forearm. The graft remains functional after 2 years [26]. There are also reports of simultaneous renal-parathyroid transplants. A young female with end-stage renal disease due to nephrocalcinosis secondary to congenital absence of parathyroid tissue received a simultaneous renal and single parathyroid transplantation from her sister. Three days following the operation, the patient demonstrated detectable levels of PTH, and the graft remained functional at 9 months post-op [27].

Different techniques have also been developed with the aim of retaining parathyroid graft endocrine function and reducing rates of graft rejection. Most surgical techniques described involve implantation of tissue into the subcutaneous fascia or muscle of the forearm. In an experimental study using rats with hypoPT, showed there was no difference in survival of parathyroid tissue implanted in sternocleidomastoid muscle, liver or peritoneum [28]. Cryopreserved parathyroid tissue which is thawed, cut into small fragments and transplanted is most commonly reported [24, 25]. Grafts made out of parathyroid tissue encapsulated into microspheres in an attempt to prevent graft rejection showed survival between 12 weeks [29] and 20 months [30]. Scaffold-free parathyroid tissue spheroids using differentiated tonsil-derived mesenchymal stem cells to restore in vivo parathyroid cell functions remained effective over 3 months [31]. It appears therefore that the limited experience reached to date has failed to identify a transplantation technique that could provide long-term clinical effectiveness.

The acknowledged limitation of this survey is its limited reach to the population of patients with hypoPT as only a quarter of the members of HypoParaUK group choose to get involved. It could be that the responders were more motivated by their severe symptoms hence their views could be biased and might not represent the entire population of patients with post-thyroidectomy hypoPT. The large number of responders suggests nevertheless that this is an important clinical topic that warrants further work in order to improve the care of patients with severely symptomatic hypoPT. The subjective nature of the study outcomes based on the self-assessment of patients is unavoidable for such studies as an objective quantifiable assessment of QoL is not feasible.

In conclusion, this study demonstrates that some patients with permanent hypoPT require a more radical and effective treatment. Majority of these patients experience severe symptoms despite regular medical treatment and their quality of life is compromised. Parathyroid transplantation is deemed by such patients to be an attractive and acceptable therapeutic option. A clinical trial should be setup to explore its feasibility and success rate.

## Electronic supplementary material

ESM 1 (PDF 393 kb)
