# Supplementary material for: Patients’ views about parathyroid transplantation for post-thyroidectomy hypoparathyroidism
Source: Langenbecks Arch Surg. 2018 Jul 3;403(5):623–9. doi: 10.1007/s00423-018-1693-y (PMC6132637; doi:10.1007/s00423-018-1693-y)
Supplement: Supplementary file 1 — (PDF 393 kb) [file 423_2018_1693_MOESM1_ESM.pdf]

PARATHYROID TRANSPLANTATION FOR TREATMENT OF POST-THYROIDECTOMY HYPOPARATHYROIDISM

Patients' needs and attitudes towards a new treatment

Part A: The severity of your condition

1. The cause of your hypoparathyroidism is...

- ◯ Injury to parathyroid glands during previous thyroid surgery
- ◯ Injury to parathyroid glands during previous parathyroid surgery
- ◯ Idiopathic (no cause known)
- ◯ Genetic syndrome

Please provide any other details you think are relevant...

2. For how many years have you suffered with this condition?

- ◯ Less than 1 year
- ◯ 1-3 years
- ◯ 3-5 years
- ◯ 5-10 years
- ◯ Over 10 years

3. In the last 6 months, how many times have you needed emergency admission to hospital for treatment of severe symptoms or extremely low calcium levels?

- ◯ Weekly
- ◯ Monthly
- ◯ Occasionally (please detail the number of visits)

4. Due to the loss of your parathyroid glands, what do you think your overall quality of life is (consider personal, social and occupational aspects)?

[1 = Worst quality of life; 9 = Best quality of life]

1	2	3	4	5	6	7	8	9
◯	◯	◯	◯	◯	◯	◯	◯	◯

5. For each of these symptoms, tell us how severe you consider them to be...

[0 = Least Severe; 5 = Most Severe]

	0	1	2	3	4	5
Numbness or tingling in hands/feet/lips	◯	◯	◯	◯	◯	◯
Muscle cramps	◯	◯	◯	◯	◯	◯
Headaches	◯	◯	◯	◯	◯	◯
Seizures	◯	◯	◯	◯	◯	◯
Bone pain	◯	◯	◯	◯	◯	◯
Fatigue	◯	◯	◯	◯	◯	◯
Generally low sense of well-being	◯	◯	◯	◯	◯	◯

6. For each of these symptoms, please tell us how often you experience them...

	Never	Intermittently	Most of the time	All of the time
Numbess or tingling sensations in hands/feet/lips	◯	◯	◯	◯
Muscle cramps	◯	◯	◯	◯
Headaches	◯	◯	◯	◯
Seizures	◯	◯	◯	◯
Bone pain	◯	◯	◯	◯
Fatigue	◯	◯	◯	◯
General sense of low well-being	◯	◯	◯	◯

7. Do you have other symptoms (i.e. not mentioned in the previous question) that could be due to hypoparathyroidism? If yes, please give details…

Please detail your daily treatment to control the low calcium levels (name of drug, dose/day)

8. Calcium supplements:

9. Vitamin D supplements:

10. Others- please detail:

11. How much do you find having to take this treatment interferes with your daily life?

[1 = Doesn't interfere with daily life; 9 = Interferes a lot with daily life]

1	2	3	4	5	6	7	8	9
○	○	○	○	○	○	○	○	○

12. In general, would you say your health is?

- ○ Excellent
- ○ Very good
- ○ Good
- ○ Fair
- ○ Poor

13. Compared to one year ago, how would you rate your health now?

- ○ Much better than one year ago
- ○ Somewhat better than one year ago
- ○ About the same as one year ago
- ○ Somewhat worse than one year ago
- ○ Much worse than one year ago

14. During the past 4 weeks, to what extent has your health (i.e. the symptoms of hypoparathyroidism) interfered with normal social activities with family, friends, neighbours, or groups?

- ○ Not at all
- ○ Slightly
- ○ Moderately
- ○ Quite a bit
- ○ Extremely

15. During the past 4 weeks, to what extent has your health interfered with your normal work (including both work outside the home and housework)?

- Not at all
- Slightly
- Moderately
- Quite a bit
- Extremely

16. Does your health now limit you in these activities? If so, how much?

	Yes, limited a lot	Yes, limited a little	No, not limited at all
Vigorous activities, such as running, heavy lifting objects or participating in strenuous sports			
Moderate activities such as moving a table, pushing a vacuum cleaner or playing golf			
Light activities such as climbing a flight of stairs, walking to shops or doing the washing-up			

Part B: Parathyroid transplantation- Current Questions

Suppose you could have a parathyroid transplant that will restore most of your parathyroid function, normalising the calcium levels in your blood and getting rid of your symptoms. However, you may have to take antirejection medication to prevent the parathyroid transplant from rejecting. These antirejection medications could have side effects and might even cause you to die earlier.

17. Choose the maximum number of years (0 to 10 years) you would be willing to give up to get a parathyroid transplant.

0	1	2	3	4	5	6	7	8	9	10
○	○	○	○	○	○	○	○	○	○	○

18. Looking at the same scenario from another angle, choose the minimum number of years you would need to live to be worth getting through the entire process of a parathyroid transplant.

0	1	2	3	4	5	6	7	8	9	10
○	○	○	○	○	○	○	○	○	○	○

19. Would you get a parathyroid transplant under any circumstance?

○ Yes

○ No

20. In all transplants there is always a chance of rejection, which could cause the complete loss of the transplanted parathyroid gland. What is the maximum chance of rejection within 1 year after the transplant that you would accept and still be willing to get the parathyroid transplant?

[90% means you would accept a high chance of rejection and 10% means you would accept a low chance of rejection]

0%	10%	20%	30%	40%	50%	60%	70%	80%	90%
○	○	○	○	○	○	○	○	○	○

21. Suppose the antirejection medications would reduce your life span by ONE THIRD.
Choose the minimum level of improvement of your quality of life that the parathyroid transplant would have to provide for you to accept such risks?

[100% means maximal improvement (identical to normal) and 10% means little improvement]

0%	10%	20%	30%	40%	50%	60%	70%	80%	90%	100%
○	○	○	○	○	○	○	○	○	○	○

22. In considering this transplant, how important is parathyroid function (i.e. freedom from symptoms) as opposed to not needing to take daily medication (i.e. freedom from medication)?

[1 = Not important; 10 = Very important]

1	2	3	4	5	6	7	8	9	10
○	○	○	○	○	○	○	○	○	○

23. If you got the parathyroid transplant, what do you think your quality of life would be (include your personal, social and occupational quality)?

[1 = Worst quality of life; 10 = Best quality of life]

1	2	3	4	5	6	7	8	9	10
○	○	○	○	○	○	○	○	○	○

Please read the below paragraph that could be part of the “patient informed consent”

You could have a parathyroid transplant that will restore most of your parathyroid function, normalising the calcium levels in your blood and getting rid of your symptoms. However, you may have to take antirejection medicines that suppress the immune system to prevent the parathyroid transplant from failing. These antirejection medications could have side effects, such as nausea, vomiting, diarrhoea, constipation, weight gain, dizziness, urinary tract infections, hypertension, diabetes, kidney failure, headaches, liver toxicity; tumours of lymph glands, skin or major organs; atherosclerosis, osteoporosis, bacterial or viral infections. Such drugs might even cause you to die earlier.

24. After reading the above would you still want to get a parathyroid transplant?

- ○ Yes
- ○ No

25. If the chance of rejecting the parathyroid gland were 50% in the first year, would you still get the parathyroid transplant?

- ○ Yes
- ○ No

26. In a few words explain which considerations would be the most important in your deciding to get, or not to get, a parathyroid transplant?

Part C: Parathyroid transplantation: Technical Aspects

There are several 'technical' options about parathyroid transplantation to be considered and it is currently not clear which one is most feasible, or with highest success rate or most likely to be approved after review by ethics committee and funding bodies.

At the present time there is no established technique or protocol for providing/attempting a parathyroid transplant. Several options could be explored, each with its own advantages and disadvantages, risks and limitations. Though this project is in its 'infancy' please give your initial thoughts on the technique involved.

27.

Option 1

Technique: *Intramuscular injection of cell suspension from a parathyroid gland removed from another patient*

Advantages: *Simple technique, likely NOT requiring long-term immunosuppressants*

Disadvantages: *Allegedly unreliable*

[1 = Certainly not interested; 5 = Certainly interested]

1	2	3	4	5
○	○	○	○	○

Please write any thoughts you may have about this option…

28.

Option 2

Technique: *Small incision over your forearm muscle to create a pocket in which to place fragments of parathyroid gland removed from another patient*

Advantages: *Acceptably simple operation*

Disadvantages: *Possible need for immunosuppressants*

[1 = Certainly not interested; 5 = Certainly interested]

1	2	3	4	5
○	○	○	○	○

Please write any thoughts you may have about this option…

29.

Option 3

Technique: *Creation of a graft formed by growing cells from a parathyroid gland into a patch of skin from the same patient, followed by a transplantation of this patch of skin over your forearm*

Advantages: *Ability to check that the graft produces parathyroid hormone before being transplanted*

Disadvantages: *Certain need for long term use of immunosuppressants*

[1 = Certainly not interested; 5 = Certainly interested]

1	2	3	4	5
○	○	○	○	○

Please write any thoughts you may have about this option…

Part D: Your involvement

30. In which way would be interested to be involved in the development of this project?

(Please tick as many as you find appropriate)

- ☐ Writing patient information leaflet to allow informed consent before the procedure
- ☐ Reviewing as a lay person the application to the Ethics Committee
- ☐ Being considered a potential candidate in the trial
- ☐ Providing support to other patients in a similar situation

Other (please specify)

31. At what email address would you like to be contacted?

Thank you for your support with this project!
